# Worker compensation injuries among the Aboriginal population of British Columbia, Canada: incidence, annual trends, and ecological analysis of risk markers, 1987–2010

**DOI:** 10.1186/1471-2458-14-710

**Published:** 2014-07-10

**Authors:** Andrew Jin, M Anne George, Mariana Brussoni, Christopher E Lalonde

**Affiliations:** 1 2762 – 133 Street, Surrey, BC V4P 1X9, Canada; 2 University of British Columbia and Child & Family Research Institute, University of Northern BC, Room 9-387, 3333 University Way, Prince George, BC V2N 3Z9, Canada; 3 University of British Columbia and Child & Family Research Institute, Child and Family Research Institute, BC Children’s Hospital, F511 - 4480 Oak Street, Vancouver, BC V6H 3 V4, Canada; 4 Department of Psychology, University of Victoria, PO Box 1700, Victoria, BC V8W 2Y2, Canada

**Keywords:** Occupational injuries (MeSH), Workers’ compensation (MeSH), Indians, North American (MeSH), Indigenous population (MeSH), “First Nations”, British Columbia (MeSH), Canada (MeSH), Epidemiology (MeSH), Population surveillance (MeSH), Socioeconomic factors (MeSH)

## Abstract

**Background:**

Aboriginal people in British Columbia (BC) have higher injury incidence than the general population, but information is scarce regarding variability among injury categories, time periods, and geographic, demographic and socio-economic groups. Our project helps fill these gaps. This report focuses on workplace injuries.

**Methods:**

We used BC’s universal health care insurance plan as a population registry, linked to worker compensation and vital statistics databases. We identified Aboriginal people by insurance premium group and birth and death record notations. We identified residents of specific Aboriginal communities by postal code. We calculated crude incidence rate and Standardized Relative Risk (SRR) of worker compensation injury, adjusted for age, gender and Health Service Delivery Area (HSDA), relative to the total population of BC. We assessed annual trend by regressing SRR as a linear function of year. We tested hypothesized associations of geographic, socio-economic, and employment-related characteristics of Aboriginal communities with community SRR of injury by multivariable linear regression.

**Results:**

During the period 1987–2010, the crude rate of worker compensation injury in BC was 146.6 per 10,000 person-years (95% confidence interval: 146.4 to 146.9 per 10,000). The Aboriginal rate was 115.6 per 10,000 (95% CI: 114.4 to 116.8 per 10,000) and SRR was 0.88 (95% CI: 0.87 to 0.89). Among those living on reserves SRR was 0.79 (95% CI: 0.78 to 0.80). HSDA SRRs were highly variable, within both total and Aboriginal populations. Aboriginal males under 35 and females under 40 years of age had lower SRRs, but older Aboriginal females had *higher* SRRs. SRRs are declining, but more slowly for the Aboriginal population. The Aboriginal population was initially at *lower* risk than the total population, but parity was reached in 2006. These community characteristics independently predicted injury risk: crowded housing, proportion of population who identified as Aboriginal, and interactions between employment rate and income, occupational risk, proportion of university-educated persons, and year.

**Conclusions:**

As employment rates rise, so has risk of workplace injury among the Aboriginal population. We need culturally sensitive prevention programs, targeting regions and industries where Aboriginal workers are concentrated and demographic groups that are at higher risk.

## Background

Aboriginal people in British Columbia (BC) have higher incidences of severe injuries (as recorded in the BC Trauma Registry) [1] or death due to injury [2-6] than the general population. However, the absolute numbers of deaths and trauma-team cases occurring among Aboriginal people in the province are small, limiting ability to break down results and make meaningful comparisons between sub-populations. This can lead to over-generalization of findings and stigmatization of Aboriginal British Columbians as a group [1]. Also, within the Aboriginal population, limited information about variability in incidence rates among injury categories, geographic regions, and demographic and socio-economic groups hampers efforts to identify risk factors and develop targeted prevention programs. The project *Injury in British Columbia’s Aboriginal Communities: Building Capacity while Developing Knowledge*[7] seeks to overcome these limitations by studying a broader range of injury morbidity events.

This report focuses on injuries claimed for worker compensation. Previous researchers in Canada have measured the incidence of worker compensation injuries among the general populations of the provinces of Ontario [8,9] and BC [10], using population-based registries [8-10] or longitudinal cohort methods [9]. Another study measured incidence, among workers in BC employed in a specific industry, by linking employment records with the injury registry [11]. The population-based studies described variations of incidence rates by gender, age, time period, and geographic location, but study of other risk markers is difficult because such information is not usually available for both individual members of the population base and individuals recorded in the injury registry. The ecological approach, where the unit of observation is a geographic unit, can help overcome this limitation, because both injury incidence, and a broad range of socio-economic, geographic, and employment-related markers can be measured at the level of the geographic unit. A previous ecologic study of predictors of risk of worker compensation injury did this among 46 regions of Ontario [12].

This report describes incidence rates, annual trends, and predictors of risk of worker compensation injury among the Aboriginal population of BC. We found no previously published report on these topics regarding the Aboriginal population of any province of Canada. We consider such information to be important to broaden the understanding of both the health status of Aboriginal British Columbians and their participation in the economic life of the province.

## Methods

### Ethics review and permission for data access

The University of British Columbia Behavioural Research Ethics Board reviewed and approved our methods. Data Stewards representing the BC Ministry of Health Services and Work Safe BC approved the data access requests. Population Data BC linked the data files and made the client records anonymous, before our analysis.

### Population counts

We obtained one-day extracts of the consolidated registration and premium billing files of the Medical Services Plan of BC (MSP, the province’s universal health care insurance program), at the mid-point of each fiscal year, 1985–1986 through 2010–2011. We took these to represent the total resident population of BC. Within this population, we marked as “Aboriginal” any person with:

a) Membership in MSP Premium Group 21 (indicating insurance premiums paid by First Nations and Inuit Health Program, Health Canada, for reason of Aboriginal status), OR

b) One or both parents with Aboriginal status or resident on an Indian Reserve, as indicated on the Vital Statistics birth record, OR

c) Aboriginal status or resident of an Indian Reserve, as indicated on the Vital Statistics death record).

For purposes of ecologic analysis (see below), within the population we identified Aboriginal “communities”. In BC there are 199 First Nations and Indian Bands recognized by and registered with the government of Canada. More than 1,000 parcels of land in BC have been designated as “reserves”, each set apart for the collective use and benefit of the members of a specified First Nation or Indian Band. Some 498 of these reserves are currently inhabited. Approximately 44% of the Aboriginal people in BC reside on a reserve (“on-reserve”) and 56% do not reside on a reserve (“off-reserve”). Conceptually, we defined a community as all the Aboriginal people residing on the reserves of one band. Operationally, we delineated each community by aggregating the postal codes of the reserves belonging to a band, and we assigned Aboriginal people to the community according to their postal code of residence. By this method, we identified 177 Aboriginal communities in BC. In fiscal year 2006–2007, total population of the communities was 62,059 and mean population per community was 351, with standard deviation of 419. The number of communities is fewer than the number of bands, because in rural areas, due to low population density, full 6-digit postal codes correspond to large areas, containing both reserves and non-reserve areas, and sometimes containing the reserves of more than one band. Thus, in practice, the identified Aboriginal communities include both Aboriginal reserve residents and off-reserve Aboriginal persons living near by, and some communities contain more than one band. Although this does not perfectly match our conceptual definition, it suffices, because it is consistent with our underlying intention, which is to identify culturally homogenous clusters of Aboriginal people living in close proximity to one another.

We aggregated the 177 identified Aboriginal communities to create a subcategory of the Aboriginal population which we called “reserve”. We classified all other Aboriginal persons as “not reserve”.

**Figure 1:**
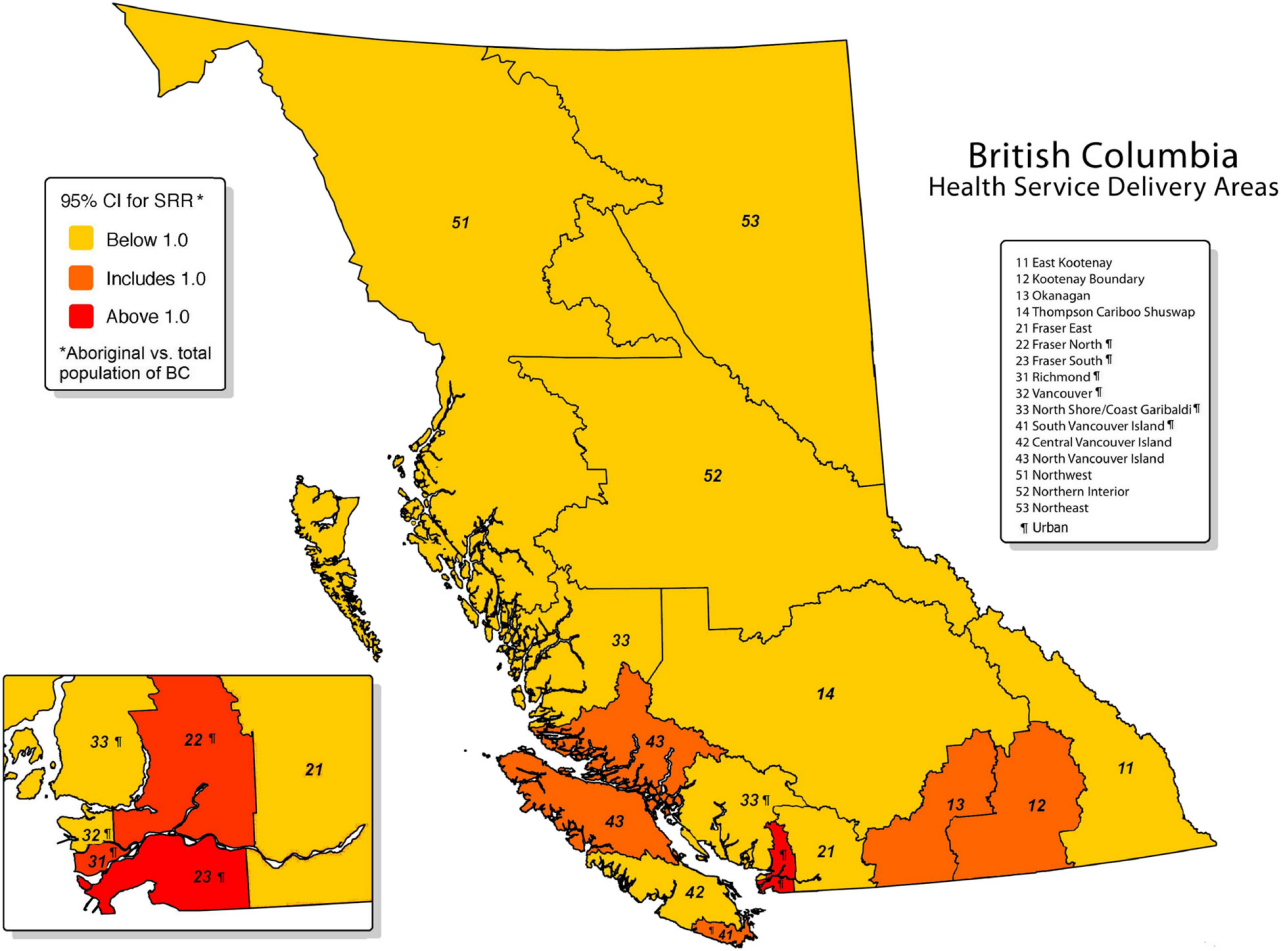
**Standardized Relative Risk of worker compensation injury among Aboriginal populations of Health Service Delivery Areas.** Adapted/reproduced with permission from the map illustration entitled "British Columbia Health Service Delivery Areas, Prepared by BC Stats, July 2008".Copyright Province of British Columbia. All Rrights reserved.

There are sixteen Health Service Delivery Areas (HSDAs) in BC. The 2011 Census of Canada found that 62.3% of the population of BC resided in urban centres with populations greater than 100,000. If more than 62.3% of the 2011 population of an HSDA resided in such an urban centre then we classified the entire HSDA (and all its residents) as “urban” [13]. In this way we classified as urban six HSDAs containing 62.7% of the 2011 population of the province [14]: HSDAs 22 (Fraser North), 23 (Fraser South), 31 (Richmond), 32 (Vancouver), 33 (North Shore/Coast Garibaldi), and 41 (South Vancouver Island). Within these six HSDAs, 88.8% of the population resided in urban centres with populations greater than 100,000. We classified all other HSDAs (and their residents) as “not urban”. Within these ten HSDAs, 17.8% of the population resided in urban centres with populations greater than 100,000. Figure 1 is a map of BC showing the 16 HSDAs in the province. The six urban HSDAs are marked with the ¶ symbol.

We tabulated population counts by fiscal year, gender, 5-year age group, Aboriginal status, community, reserve residence, HSDA, and urban residence.

### Worker compensation injuries

We tabulated counts of worker compensation injuries among residents of BC, occurring from January 1, 1987 through December 31, 2010. We defined “worker compensation injury” as an injury registered for a claim with Work Safe BC (the province’s workplace injury compensation program), with an ICD-9 numeric code diagnosis in the range 800 through 999. This definition excludes some chronic conditions recognized as injuries by Work Safe BC, for example, tendonitis, carpal tunnel syndrome, noise-induced hearing loss, occupational lung diseases, and occupational cancers. Work Safe BC provides compensation for injury or disease that arises out of and in the course of employment, or is due to the nature of employment. Employers are required by law to register with Work Safe BC to provide coverage to their employees. Aboriginal subsistence activities (e.g., hunting, fishing, trapping, gathering wild plants, cutting trees) may be covered, if the individual registers with Work Safe BC and pays insurance premiums for the optional personal protection available to self-employed persons. In Canada, Aboriginal subsistence includes a right to earn a moderate living by selling the products of one’s labour. Unpaid domestic labour is not considered employment. Injury occurring while travelling between one’s place of residence and place of employment does not meet the test of “arising out of and in the course of employment, or due to the nature of employment”. Full-time or part-time labour does not influence acceptance of an injury claim, though it does influence the amount of compensation.

We classified worker compensation injuries by injury type (trauma, poisoning, burn or other) using ranges of the ICD-9 numeric code diagnosis. We tabulated counts of injuries by injury type, calendar year (of injury occurrence), gender, 5-year age group, Aboriginal status, reserve residence, HSDA, and urban residence.

### Incidence rates of injury

We calculated the crude rate of worker compensation injuries as the number of injuries divided by the person-years of observation (the sum of the annual population counts) during the same time period. We considered the crude rate to be a binomial proportion, and we estimated standard errors of the proportion, and 95% confidence intervals of the proportion, using the method of Agresti and Coull [15]. Consistent with Statistics Canada policy [16,17], we suppressed reporting of the crude rate in a cell if the coefficient of variation (the standard error of the crude rate divided by the crude rate) exceeded 0.333.

We calculated rates of worker compensation injury using person-years of *population* as the denominator, because we consider such rates to be indicators of population health status (limited to one specific category of health outcome). Other researchers have used person-years of *employment* as the rate denominator, which would be appropriate if one thinks of injury risk in the manner of an insurer seeking to justify premiums levied on employers according to the size of the workforce. But that was not our intention. Also, our population counts are more reliable than estimates of numbers of employed persons derived from survey samples, which would also have had to be adjusted for intensity of employment (i.e., full-time or part-time employment) with even more propagation of random measurement error.

We calculated **Standardized Relative Risk** (SRR) of worker compensation injury relative to the risk of injury in the reference population (95,457,166 person-years, the combined total population of BC from January 1, 1987 through December 31, 2010) using the method of indirect standardization [18], adjusting for gender and age, or gender, age and HSDA, as appropriate for the intended comparisons. We suppressed reporting of the SRR in a cell if the coefficient of variation (the standard error of the expected number of injuries divided by the expected number) exceeded 0.333.

**Figure 2:**
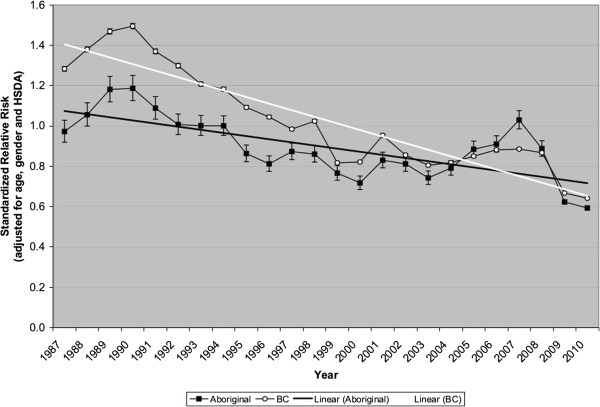
Worker compensation injuries, British Columbia, 1987–2010, Standardized Relative Risk by year.

The error bars in Figure 2 depict 95% confidence intervals. Comparing two crude rates or two SRRs, we considered the difference to be “statistically significant” if the 95% confidence intervals did not overlap. This indicates p < 0.006, if the standard errors are equal, or p < 0.021 if one of the standard errors is up to five times larger than the other [19].

We assessed annual trend as a linear function with year as the independent variable, and SRR as the dependent variable. We considered the trend to be “statistically significant” if the 95% confidence interval of the regression coefficient (the slope) did not include zero.

### Predictors of risk

We expected that the individual-level analysis methods above would describe heterogeneity among age and gender groups, among geographic regions, among fiscal years of observation, between Aboriginal and non-Aboriginal, and between on-reserve Aboriginal and off-reserve Aboriginal populations, but would not explain why the heterogeneities exist. Therefore, to elucidate possible explanatory factors, we studied risk markers for worker compensation injury among the Aboriginal population using an ecological approach, where the unit of observation was the “community” (as defined above). As hypothesized risk factors, we selected socio-economic, housing, and geographic indicators that had previously been developed by Statistics Canada and Aboriginal Affairs and Northern Development Canada, which are used to allocate federal government resources to health care, education, housing, and economic development programs for Aboriginal people. We wanted to test if these markers had predictive validity with respect to risk of worker compensation injury, which is indicative of both health status and economic development.

Within communities, risk of injury among Aboriginal people is calculated using our own definition of “Aboriginal”, derived from health insurance premium group and notations on birth and death records. However, every First Nation band makes its own residency rules, and not all residents of reserves would meet our definition of Aboriginal. We wanted to test if variability in the ethnic composition of reserve populations would introduce biases into our ecologic analysis, and if so, to correct such biases. Therefore, we included in the analysis two Census-derived ecologic indicators describing ethnic composition.

From the 2001 and the 2006 Censuses of Canada we obtained customized data tabulations for all enumerated First Nation reserves, settlements or self-government districts in BC, aggregated by First Nation band. The Census long-form (usually administered to a 20% sample of the population) was administered to 100% of residents of First Nation reserves, settlements and self-government districts. From these data, for as many communities as possible, we tabulated the following hypothesized socio-economic markers of injury risk:

•Total Income per capita,

•Community Well-Being Income Score [20], calculated as: Log_10_[(Total Income per capita)/2000] / Log_10_[20] × 100,

•Proportion of population, age 25+ years with at least a high school certificate,

•Proportion of population, age 25+ years with university degree, bachelors or higher,

•Average population per room (an index of the degree of crowding in the community’s housing), calculated as the number of residents divided by the number of habitable rooms (not counting bathrooms, halls, vestibules and rooms used solely for business purposes) in the dwelling,

•Proportion of dwellings in need of major repairs (defective plumbing or electrical wiring, structural repairs to walls, floors or ceilings, etc., does not include desirable remodelling or additions),

•Proportion of population, age 25+ years, in the labour force (in the week before the census, employed, temporarily absent, looking for work, or starting work within 4 weeks),

•Proportion of population, age 25+ years, employed (any work for pay or self-employment in the week before the census),

•Proportion of population who identified themselves as “an Aboriginal person, that is, North American Indian, Métis or Inuit (Eskimo)”,

•Proportion of population who gave only one response to the ethnic origin question, and it was a group that could be classified as North American Indian.

Some calculated proportions exceeded 100% because Statistics Canada rounds cell counts to the nearest multiple of five, to protect privacy. If a community contained more than one First Nation band, then we calculated the community’s marker as the population-weighted mean of the First Nation bands’ markers. Statistics Canada reports only the total population count for aggregations with population less than 40, and suppresses income data for aggregations with population less than 250. We were able to calculate the two income-related markers for 79 (of 177) communities in Census year 2001, and 73 communities in 2006. We were able to calculate the other markers listed above for 151 communities in 2001, and 127 communities in 2006.

Rates of worker compensation claims differ among occupational [21] and industrial categories [22], and these factors (and size of payroll and previous claims experience) determine the insurance premiums that Work Safe BC levies upon employers. We hypothesized that the distribution of the community’s labour force among occupational and industrial categories would help explain the community’s risk of worker compensation injury. We invented two statistics that summarize the hypothesized hazardousness of the community’s labour force distribution. Each statistic is the mean risk of work injury claim among the occupational or industrial categories in the total population of BC, weighted by the number of persons in each occupational or industrial category in the community. Combining Work Safe BC injury claims statistics and Census data, we calculated the following work-related statistics of injury risk for each community:

•Risk of work injury claim, relative to the population of BC, expected from occupational categories [21], among labour force aged 15+ years,

•Risk of work injury claim, relative to the population of BC, expected from industry categories [22], among labour force aged 15+ years.

The Government of Canada’s Department of Aboriginal Affairs and Northern Development has a classification system for calculating funding allocations to First Nation bands [23]. From this system, we assigned to communities the following hypothesized geographic markers of injury risk:

•Remoteness Index (higher score means more remote), and

•Environmental Index (higher score means more environmentally challenging).

These indices are numeric scores, based on geographic latitude, availability of year-round road access, and distance to the nearest “service centre” (a city or town having government services, banks and suppliers). If a community contained more than one First Nation band, then we calculated the community’s index as the population-weighted mean of the bands’ indices.

Worker compensation injury can only occur to employed people. It is plausible that risk factors for such injury would apply only to the fraction of the population who are employed. Therefore, for each of the above hypothesized socio-economic, work-related, and geographic risk markers we also created an employment-interaction variable, calculated as the risk marker multiplied by the proportion of the population in each community who were employed.

### Ecological analysis

For each community, we calculated the age, gender and HSDA-adjusted SRR of worker compensation injury during the period 1999 through 2003 (a 5-year period centred about the Census year 2001) and during the period 2004 through 2008 (centred about the Census year 2006), relative to the total population of BC during the same time period. Logarithmic transformation approximately normalized the distribution of the SRRs (Kolmogorov-Smirnov statistic 0.058, Shapiro-Wilk statistic 0.988, df = 319, p = 0.012); therefore we used the natural logarithm of SRR as the dependent (Y) variable for regression analysis.

We tested hypotheses of association by performing least-squares linear regressions. We tested census year, hypothesized socio-economic, work-related and geographic markers, and their employment-interaction variables, in turn as the single independent variable. Variables that had statistically significant association (p < 0.05) with SRR of worker compensation injury in univariate analysis were included in subsequent multivariable regression analysis. We used stepwise backwards elimination of variables to arrive at the best-fitting multivariable model. At each step, the variable with the largest p-value was eliminated. Elimination stopped when all independent variables had regression coefficients significantly different from zero (p < 0.05).

In the best-fitting model, “B” is the regression coefficient of each independent variable, representing the change in the dependent variable Ln (SRR) that is associated with a unit change in the independent variable. The relative risk associated with a *one standard deviation* change (SD) in the independent variable is calculated as the antilogarithm of BxSD. Repeating the calculation with the lower and upper 95% confidence limits of B gives the confidence limits of the relative risk.

## Results

### Aboriginal status and reserve residence

Table 1 shows crude rates and SRRs of injuries claimed for worker compensation, during the period 1987–2010, among the total population of BC, the Aboriginal population, the Aboriginal population residing on reserve, and the Aboriginal population residing off-reserve. Table 1 also separates injuries into broad ranges of the ICD-9 numeric classification: trauma, poisoning, burn, and other. Because 96% of worker compensation injuries are in the category of trauma, we combined all injury categories for the remainder of the description and analysis.

**Table 1:** Worker compensation injuries [1], British Columbia, 1987–2010 [2]

**Injury Category [3]**		**P-years [4]**	**Obs [5]**	**Exp [6]**	**Rate [7]**	**95% CI for Rate**			**SRR [8]**	**95% CI for SRR**		
**BC**												
	**Total, All injuries**	**95,457,166**	**1,399,661**	**1,399,659**	**146.6**	**146.4**	**to**	**146.9**	**1.00**	**1.00**	**to**	**1.00**
	. Trauma	95,457,166	1,343,044	1,343,042	140.7	140.5	to	140.9	1.00	1.00	to	1.00
	. Poisoning	95,457,166	6,469	6,469	0.7	0.7	to	0.7	1.00	0.98	to	1.02
	. Burn	95,457,166	45,612	45,612	4.8	4.7	to	4.8	1.00	0.99	to	1.01
	. Other	95,457,166	4,536	4,536	0.5	0.5	to	0.5	1.00	0.97	to	1.03
**BC, Aboriginal**												
	**Total, All injuries**	**3,091,021**	**35,736**	**40,608**	**115.6**	**114.4**	**to**	**116.8**	**0.88**	**0.87**	**to**	**0.89**
	. Trauma	3,091,021	34,504	38,826	111.6	110.5	to	112.8	0.89	0.88	to	0.90
	. Poisoning	3,091,021	180	202	0.6	0.5	to	0.7	0.89	0.78	to	1.02
	. Burn	3,091,021	903	1,429	2.9	2.7	to	3.1	0.63	0.60	to	0.67
	. Other	3,091,021	149	151	0.5	0.4	to	0.6	0.99	0.84	to	1.16
**BC, Aboriginal, off-reserve**												
	**Total, All injuries**	**1,688,590**	**20,983**	**21,898**	**124.3**	**124.3**	**to**	**122.6**	**0.96**	**0.95**	**to**	**0.97**
	. Trauma	1,688,590	20,202	20,931	119.6	119.6	to	118.0	0.97	0.95	to	0.98
	. Poisoning	1,688,590	98	106	0.6	0.6	to	0.5	0.92	0.76	to	1.12
	. Burn	1,688,590	597	781	3.5	3.5	to	3.3	0.76	0.71	to	0.82
	. Other	1,688,590	86	79	0.5	0.5	to	0.4	1.08	0.87	to	1.35
**BC, Aboriginal, on-reserve**												
	**Total, All injuries**	**1,393,652**	**14,641**	**18,595**	**105.1**	**105.1**	**to**	**103.4**	**0.79**	**0.78**	**to**	**0.80**
	. Trauma	1,393,652	14,195	17,787	101.9	101.9	to	100.2	0.80	0.79	to	0.81
	. Poisoning	1,393,652	81	95	0.6	0.6	to	0.5	0.85	0.70	to	1.04
	. Burn	1,393,652	302	643	2.2	2.2	to	1.9	0.47	0.43	to	0.51
	. Other	1,393,652	63	71	0.5	0.5	to	0.4	0.89	0.70	to	1.13

Notes:

1. “Injury” defined as Diagnosis in the range ICD9:800–999.

2. Injuries occurring during the observation period 1987-Jan-01 to 2010-Dec-31.

3. Injuries classified by ICD9 numeric code.

4. Person-years is the sum of annual population counts during the observation period.

5. Observed number of injuries.

6. Expected number, indirectly standardized, based on age, gender and HSDA-specific rates in the total population of BC.

7. Crude Rate per 10,000 person-years.

8. Standardized Relative Risk (compared to the total population of BC) = Observed/Expected.

Table 1 shows a pattern of the lowest incidence among the Aboriginal population on or near a reserve, higher incidence among the Aboriginal population off-reserve, and highest incidence in the total population of BC. Standardization by age, gender and HSDA reduces but does not eliminate the disparities among the three population groups. In particular, the gap between the off-reserve Aboriginal population and the total population of BC (i.e., the reference population) is small, but remains statistically significant.

### HSDAs and urban residence

Tables 2 and 3 show crude rates and age and gender-adjusted SRRs of injuries claimed for worker compensation, during the period 1987–2010, within the total populations (Table 2) and the Aboriginal populations (Table 3) of the HSDAs. Depending on the HSDA, Aboriginal people may be at higher (SRR > 1), lower (SRR < 1), or the same risk (SRR = 1) of injury as the total population of the province (Figure 1). There are differences in risk of worker compensation injury between HSDAs, but these differences do not necessarily apply to both the Aboriginal and the total populations. For example, within the total population, the highest age- and gender-standardized risks of worker compensation injury occur in HSDAs 21, 22 and 23, but within the Aboriginal population, the highest risks occur in HSDAs 22, 23 and 31. Within the total population, urban and not urban residents had the same age- and gender-standardized risks of worker compensation injury, but within the Aboriginal population, urban residents had higher age- and gender-standardized risk of worker compensation injury (SRR = 0.95, 95% confidence interval: 0.93 to 0.96) than those who were not urban (SRR = 0.79, 95% CI: 0.78 to 0.80). However, as shown in Table 3 and Figure 1, not all urban HSDAs showed above-average risks among their Aboriginal populations: HSDAs 22, 23 and 31 did (lower 95% confidence limit of SRR was above one), but HSDAs 32 and 33 clearly did not (upper 95% confidence limit of SRR was below one).

**Table 2:** Worker compensation injuries [1], British Columbia, 1987–2010 [2], by Health Service Delivery Area

**HSDA**	**P-years [3]**	**Obs [4]**	**Exp [5]**	**Rate [6]**	**95% CI for Rate**			**SRR [7]**	**95% CI for SRR**		
11	1,847,429	22,605	25,976	122	121	to	124	0.87	0.86	to	0.88
12	1,878,968	24,204	25,856	129	127	to	130	0.94	0.92	to	0.95
13	7,129,280	92,766	93,549	130	129	to	131	0.99	0.99	to	1.00
14	4,987,600	59,730	70,736	120	119	to	121	0.84	0.84	to	0.85
21	5,455,829	94,435	74,278	173	172	to	174	1.27	1.26	to	1.28
22	11,998,748	211,048	183,029	176	175	to	177	1.15	1.15	to	1.16
23	13,344,187	251,995	191,340	189	188	to	190	1.32	1.31	to	1.32
31	3,979,078	51,080	59,421	128	127	to	129	0.86	0.85	to	0.87
32	13,897,287	170,380	224,694	123	122	to	123	0.76	0.76	to	0.76
33	6,104,957	69,383	88,384	114	113	to	114	0.79	0.78	to	0.79
41	7,873,455	104,328	109,547	133	132	to	133	0.95	0.95	to	0.96
42	5,507,969	77,846	73,643	141	140	to	142	1.06	1.05	to	1.06
43	2,656,173	42,981	37,237	162	160	to	163	1.15	1.14	to	1.17
51	2,034,014	28,103	30,192	138	137	to	140	0.93	0.92	to	0.94
52	3,562,522	44,853	53,539	126	125	to	127	0.84	0.83	to	0.84
53	1,551,472	17,162	23,369	111	109	to	112	0.73	0.73	to	0.74
Urban [8]	57,197,712	858,214	856,415	150	150	to	150	1.00	1.00	to	1.00
Not [9]	36,611,256	504,685	508,375	138	137	to	138	0.99	0.99	to	1.00

Notes:

1. “Injury” defined as any diagnosis in the range ICD9:800–999

2. Injuries occurring during the observation period 1987-Jan-01 to 2010-Dec-31.

3. Person-years is the sum of annual population counts during the observation period.

4. Observed number of injuries.

5. Expected number, indirectly standardized, based on age- and gender-specific rates in total population of BC.

6. Crude Rate per 10,000 person-years.

7. Standardized Relative Risk (compared to the total population of BC) = Observed/Expected.

8. Urban: aggregation of HSDAs 22, 23, 31, 32, 33 and 41, where > 62.3% of the HSDA population live in a large population centre.

9. Not urban: aggregation of HSDAs 11, 12, 13, 14, 21, 42, 43, 51, 52, 53.

**Table 3:** Worker compensation injuries [1], Aboriginal BC, 1987–2010 [2], by Health Service Delivery Area

**HSDA**	**P-years [3]**	**Obs [4]**	**Exp [5]**	**Rate [6]**	**95% CI for Rate**			**SRR [7]**	**95% CI for SRR**		
11	38,313	443	512	116	105	to	127	0.87	0.79	to	0.94
12	13,647	165	169	121	104	to	141	0.98	0.84	to	1.14
13	161,664	2,177	2,184	135	129	to	140	1.00	0.96	to	1.04
14	404,410	3,821	5,738	94	92	to	98	0.67	0.65	to	0.68
21	196,605	2,393	2,612	122	117	to	127	0.92	0.88	to	0.95
22	111,440	1,967	1,526	177	169	to	184	1.29	1.23	to	1.36
23	122,044	1,927	1,437	158	151	to	165	1.34	1.27	to	1.41
31	17,062	404	229	237	215	to	261	1.76	1.55	to	2.01
32	261,269	2,916	4,152	112	108	to	116	0.70	0.68	to	0.72
33	233,561	2,868	3,360	123	118	to	127	0.85	0.83	to	0.88
41	156,312	2,090	2,160	134	128	to	140	0.97	0.93	to	1.01
42	329,123	3,711	4,353	113	109	to	116	0.85	0.83	to	0.88
43	157,943	2,108	2,130	133	128	to	139	0.99	0.95	to	1.03
51	490,310	5,348	6,854	109	106	to	112	0.78	0.76	to	0.80
52	275,145	2,279	3,706	83	80	to	86	0.61	0.60	to	0.63
53	98,686	846	1,277	86	80	to	92	0.66	0.63	to	0.70
Urban [8]	901,688	12,172	12,864	135	133	to	137	0.95	0.93	to	0.96
Not [9]	2,165,846	23,291	29,534	108	106	to	109	0.79	0.78	to	0.80

Notes:

1. “Injury” defined as any diagnosis in the range ICD9:800–999.

2. Injuries occurring during the observation period 1987-Jan-01 to 2010-Dec-31.

3. Person-years is the sum of annual population counts during the observation period.

4. Observed number of injuries.

5. Expected number, indirectly standardized, based on age- and gender-specific rates in total population of BC.

6. Crude Rate per 10,000 person-years.

7. Standardized Relative Risk (compared to the total population of BC) = Observed/Expected.

8. Urban: aggregation of HSDAs 22, 23, 31, 32, 33 and 41, where > 62.3% of the HSDA population live in a large population centre.

9. Not urban: aggregation of HSDAs 11, 12, 13, 14, 21, 42, 43, 51, 52, 53.

### Age and gender

Tables 4 and 5 show crude rates and SRRs of injuries claimed for worker compensation, among the total population (Table 4) and the Aboriginal population (Table 5) of BC, by age and gender categories. Crude rates (age- and gender-specific) of worker compensation injury are higher among males than among females in all age groups. Among males, injury rates are highest among men aged 20 to 29 years, and decline steadily as age increases. Among females, worker compensation injury rates are highest among women aged 40 to 54 years.

**Table 4:** Worker compensation injuries [1], British Columbia, 1987–2010 [2], by Age and Gender

**Gender**	**Age**	**P-years [3]**	**Obs [4]**	**Exp [5]**	**Rate [6]**	**95% CI for Rate**			**SRR [7]**	**95% CI for SRR**		
F	15-19	3,091,296	18,913	18,913	61	60	to	62	1.00	0.99	to	1.01
F	20-24	3,215,407	39,379	39,379	122	121	to	124	1.00	0.99	to	1.01
F	25-29	3,478,049	42,537	42,537	122	121	to	123	1.00	0.99	to	1.01
F	30-34	3,702,923	45,675	45,675	123	122	to	124	1.00	0.99	to	1.01
F	35-39	3,861,158	50,660	50,660	131	130	to	132	1.00	0.99	to	1.01
F	40-44	3,830,469	53,141	53,141	139	138	to	140	1.00	0.99	to	1.01
F	45-49	3,525,752	50,340	50,340	143	142	to	144	1.00	0.99	to	1.01
F	50-54	3,024,714	40,801	40,801	135	134	to	136	1.00	0.99	to	1.01
F	55-59	2,558,851	26,963	26,963	105	104	to	107	1.00	0.99	to	1.01
F	60-64	2,186,965	11,050	11,050	51	50	to	51	1.00	0.98	to	1.02
F	65-69	1,912,893	1,279	1,279	7	6	to	7	1.00	0.95	to	1.06
F	70-74	1,670,886	225	225	1	1	to	2	1.00	0.88	to	1.14
M	15-19	3,256,059	51,250	51,250	157	156	to	159	1.00	0.99	to	1.01
M	20-24	3,186,968	137,742	137,742	432	430	to	434	1.00	0.99	to	1.01
M	25-29	3,377,301	154,893	154,893	459	456	to	461	1.00	1.00	to	1.00
M	30-34	3,611,964	155,457	155,457	430	428	to	432	1.00	1.00	to	1.00
M	35-39	3,797,595	142,295	142,295	375	373	to	377	1.00	0.99	to	1.01
M	40-44	3,806,541	122,056	122,056	321	319	to	322	1.00	0.99	to	1.01
M	45-49	3,542,795	97,188	97,188	274	273	to	276	1.00	0.99	to	1.01
M	50-54	3,056,634	73,977	73,977	242	240	to	244	1.00	0.99	to	1.01
M	55-59	2,591,967	51,784	51,784	200	198	to	201	1.00	0.99	to	1.01
M	60-64	2,179,698	25,923	25,923	119	117	to	120	1.00	0.99	to	1.01
M	65-69	1,821,029	4,182	4,182	23	22	to	24	1.00	0.97	to	1.03
M	70-74	1,479,753	1,024	1,024	7	7	to	7	1.00	0.94	to	1.06

Notes:

1. “Injury” defined as any diagnosis in the range ICD9:800–999.

2. Injuries occurring during the observation period 1987-Jan-01 to 2010-Dec-31.

3. Person-years is the sum of annual population counts during the observation period.

4. Observed number of injuries.

5. Expected number, indirectly standardized, based on age, gender and HSDA-specific rates in total population of BC.

6. Crude Rate per 10,000 person-years.

7. Standardized Relative Risk (compared to the total population of BC) = Observed/Expected.

**Table 5:** Worker compensation injuries [1], Aboriginal BC, 1987–2010 [2], by Age and Gender

**Gender**	**Age**	**P-years [3]**	**Obs [4]**	**Exp [5]**	**Rate [6]**	**95% CI for Rate**			**SRR [7]**	**95% CI for SRR**		
F	15-19	135,848	385	790	28	26	to	31	0.49	0.45	to	0.52
F	20-24	127,128	890	1,445	70	66	to	75	0.62	0.58	to	0.65
F	25-29	129,776	1,098	1,497	85	80	to	90	0.73	0.70	to	0.77
F	30-34	129,199	1,317	1,496	102	97	to	108	0.88	0.84	to	0.93
F	35-39	122,522	1,405	1,522	115	109	to	121	0.92	0.88	to	0.97
F	40-44	108,519	1,397	1,419	129	122	to	136	0.98	0.93	to	1.04
F	45-49	89,472	1,175	1,192	131	124	to	139	0.99	0.93	to	1.04
F	50-54	68,418	865	846	126	118	to	135	1.02	0.96	to	1.09
F	55-59	51,343	589	488	115	106	to	124	1.21	1.10	to	1.32
F	60-64	38,327	226	176	59	52	to	67	1.28	1.11	to	1.49
F	65-69	27,860	29	20	10	7	to	15	1.45	0.93	to	2.28
F	70-74	19,479	7	3	4	2	to	8	2.66	0.80	to	19.00
M	15-19	138,807	1,117	2,012	80	76	to	85	0.56	0.53	to	0.58
M	20-24	122,074	3,753	5,001	307	298	to	317	0.75	0.73	to	0.77
M	25-29	124,279	4,807	5,552	387	376	to	398	0.87	0.84	to	0.89
M	30-34	122,053	4,834	5,073	396	385	to	407	0.95	0.93	to	0.98
M	35-39	114,734	4,095	4,115	357	346	to	368	1.00	0.97	to	1.03
M	40-44	100,165	3,026	3,066	302	292	to	313	0.99	0.95	to	1.02
M	45-49	81,440	2,063	2,130	253	243	to	264	0.97	0.93	to	1.01
M	50-54	61,645	1,416	1,412	230	218	to	242	1.00	0.95	to	1.06
M	55-59	46,051	787	853	171	159	to	183	0.92	0.86	to	0.99
M	60-64	33,729	339	379	101	90	to	112	0.89	0.81	to	0.99
M	65-69	24,066	65	61	27	21	to	34	1.06	0.83	to	1.37
M	70-74	16,365	16	12	10	6	to	16	1.28	0.73	to	2.30

Notes:

1. “Injury” defined as any diagnosis in the range ICD9:800–999.

2. Injuries occurring during the observation period 1987-Jan-01 to 2010-Dec-31.

3. Person-years is the sum of annual population counts during the observation period.

4. Observed number of injuries.

5. Expected number, indirectly standardized, based on age, gender and HSDA-specific rates in total population of BC.

6. Crude Rate per 10,000 person-years.

7. Standardized Relative Risk (compared to the total population of BC) = Observed/Expected.

SRRs (adjusted for age, gender and HSDA) show that younger Aboriginal persons (males under 35 years and females under 40 years of age) have lower risk of worker compensation injury compared to persons of the same age and gender in the total population. Older Aboriginal males have about the same risk of worker compensation injury as males in the total population. Older Aboriginal females have *higher* risk of worker compensation injury than females in the total population.

### Annual trends

Tables 6 and 7 show crude rates and SRRs of injuries claimed for worker compensation, during the period 1987–2010, among the total population (Table 6) and the Aboriginal population (Table 7), by year. Figure 2 depicts comparisons of SRRs between these populations, regarding all injuries combined. SRRs in both the tables and figures have been adjusted for age, gender, and HSDA. Recall that the reference population is the combined total population of BC during the entire period (1987 through 2010). Thus, the SRR for the total population of BC in a particular year can be higher or lower than one, but the average of the SRRs for the total population of BC, over all the years, will be one.

**Table 6:** Worker compensation injuries [1], British Columbia, 1987–2010 [2], by Year

**Year**	**P-years [3]**	**Obs [4]**	**Exp [5]**	**Rate [6]**	**95% CI for Rate**			**SRR [7]**	**95% CI for SRR**		
1987	3,121,318	56,943	44,364	182	181	to	184	1.28	1.27	to	1.30
1988	3,165,022	62,293	45,153	197	195	to	198	1.38	1.37	to	1.39
1989	3,245,277	68,314	46,509	211	209	to	212	1.47	1.46	to	1.48
1990	3,339,763	72,124	48,247	216	214	to	218	1.49	1.48	to	1.51
1991	3,421,459	67,786	49,492	198	197	to	200	1.37	1.36	to	1.38
1992	3,515,345	66,197	50,970	188	187	to	190	1.30	1.29	to	1.31
1993	3,649,925	64,624	53,506	177	176	to	178	1.21	1.20	to	1.22
1994	3,771,519	65,575	55,449	174	173	to	175	1.18	1.17	to	1.19
1995	3,856,183	61,873	56,649	160	159	to	162	1.09	1.08	to	1.10
1996	3,959,300	60,944	58,338	154	153	to	155	1.04	1.04	to	1.05
1997	4,040,687	58,697	59,628	145	144	to	146	0.98	0.98	to	0.99
1998	4,087,714	61,689	60,256	151	150	to	152	1.02	1.02	to	1.03
1999	4,115,601	49,584	60,640	120	119	to	122	0.82	0.81	to	0.82
2000	4,114,815	49,678	60,464	121	120	to	122	0.82	0.82	to	0.83
2001	4,160,615	58,317	61,161	140	139	to	141	0.95	0.95	to	0.96
2002	4,211,443	52,979	61,890	126	125	to	127	0.86	0.85	to	0.86
2003	4,285,095	50,983	63,310	119	118	to	120	0.81	0.80	to	0.81
2004	4,335,962	52,636	64,324	121	120	to	122	0.82	0.81	to	0.82
2005	4,383,639	55,255	65,001	126	125	to	127	0.85	0.84	to	0.86
2006	4,414,528	57,497	65,192	130	129	to	131	0.88	0.88	to	0.89
2007	4,476,436	58,476	66,025	131	130	to	132	0.89	0.88	to	0.89
2008	4,546,001	58,183	67,066	128	127	to	129	0.87	0.86	to	0.87
2009	4,607,365	45,298	67,875	98	97	to	99	0.67	0.66	to	0.67
2010	4,632,154	43,716	68,149	94	93	to	95	0.64	0.64	to	0.65

**Notes**:

1. “Injury” defined as any diagnosis in the range ICD9:800–999.

2. Injuries occurring during the observation period 1987-Jan-01 to 2010-Dec-31.

3. Person-years is the population count during the specified year.

4. Observed number of injuries.

5. Expected number, indirectly standardized, based on age, gender and HSDA-specific rates in total population of BC during entire observation period.

6. Crude Rate per 10,000 person-years.

7. Standardized Relative Risk (compared to the total population of BC during the entire observation period) = Observed/Expected.

**Table 7:** Worker compensation injuries [1], Aboriginal BC, 1987–2010 [2], by Year

**Year**	**P-years [3]**	**Obs [4]**	**Exp [5]**	**Rate [6]**	**95% CI for Rate**			**SRR [7]**	**95% CI for SRR**		
1987	96,252	1,174	1,207	122	115	to	129	0.97	0.92	to	1.03
1988	99,507	1,336	1,266	134	127	to	142	1.06	1.00	to	1.11
1989	102,607	1,567	1,327	153	145	to	160	1.18	1.12	to	1.25
1990	104,866	1,639	1,381	156	149	to	164	1.19	1.13	to	1.25
1991	108,471	1,564	1,437	144	137	to	151	1.09	1.03	to	1.15
1992	111,758	1,499	1,489	134	128	to	141	1.01	0.96	to	1.06
1993	116,061	1,560	1,558	134	128	to	141	1.00	0.95	to	1.05
1994	119,614	1,609	1,608	135	128	to	141	1.00	0.95	to	1.05
1995	122,026	1,416	1,640	116	110	to	122	0.86	0.82	to	0.91
1996	124,891	1,365	1,681	109	104	to	115	0.81	0.77	to	0.85
1997	126,909	1,488	1,704	117	111	to	123	0.87	0.83	to	0.92
1998	128,332	1,478	1,718	115	109	to	121	0.86	0.82	to	0.90
1999	128,945	1,318	1,720	102	97	to	108	0.77	0.73	to	0.80
2000	130,683	1,243	1,732	95	90	to	101	0.72	0.68	to	0.75
2001	133,025	1,457	1,755	110	104	to	115	0.83	0.79	to	0.87
2002	135,727	1,446	1,781	107	101	to	112	0.81	0.78	to	0.85
2003	139,955	1,370	1,845	98	93	to	103	0.74	0.71	to	0.78
2004	142,881	1,485	1,877	104	99	to	109	0.79	0.76	to	0.83
2005	145,834	1,687	1,907	116	110	to	121	0.88	0.85	to	0.92
2006	148,458	1,759	1,932	118	113	to	124	0.91	0.87	to	0.95
2007	151,609	2,023	1,964	133	128	to	139	1.03	0.99	to	1.08
2008	154,876	1,769	1,993	114	109	to	120	0.89	0.85	to	0.93
2009	158,252	1,265	2,030	80	76	to	84	0.62	0.60	to	0.65
2010	159,482	1,219	2,055	76	72	to	81	0.59	0.57	to	0.62

**Notes**:

1. “Injury” defined as any diagnosis in the range ICD9:800–999.

2. Injuries occurring during the observation period 1987-Jan-01 to 2010-Dec-31.

3. Person-years is the population count during the specified year.

4. Observed number of injuries.

5. Expected number, indirectly standardized, based on age, gender and HSDA-specific rates in total population of BC during entire observation period.

6. Crude Rate per 10,000 person-years.

7. Standardized Relative Risk (compared to the total population of BC during the entire observation period) = Observed/Expected.

SRR trends (Figure 2) show that risks of injury are declining, although the rate of decline has been greater for the total population (mean change in SRR was −0.033 per year, 95% confidence interval: −0.039 to −0.027) than for the Aboriginal population (mean change in SRR was −0.016 per year, 95% CI: −0.022 to −0.009). The Aboriginal population was at *lower* risk than the total population at the start of the period (1987), but parity was reached (the trend lines converged) in 2006. The risk of worker compensation injury among the Aboriginal population increased during the years 2003 through 2007, then declined markedly.

### Ecological analysis of predictors of risk

Our analysis of custom data from the Census showed that Aboriginal people residing on reserves have lower employment rates than the total population of BC (45.4% vs. 61.1% in 2001, and 46.2% vs. 62.4% in 2006); on the other hand, when they are employed, they are more likely to work in hazardous occupations (expected relative risk of worker compensation claim, “RR” was 1.10 in 2001, increasing to 1.14 in 2006) or industries (RR = 1.08 in both 2001 and 2006). Compared to the male labour force of BC, the Aboriginal male labour force residing on reserves are more concentrated in “trades, transport and equipment operators and related occupations”, “occupations unique to primary industry”, and “occupations unique to processing, manufacturing and utilities” (i.e., the proportion of the Aboriginal labour force in each of these categories was greater than the proportion of the BC general population labour force in the same category.) These are “blue-collar” occupational groups, with relatively higher rates of worker compensation claims [21]. The Aboriginal male labour force is also more concentrated in “occupations in social science, education, government service and religion.” This is generally an occupational category with a low risk of worker compensation claim [21]. However, on Aboriginal reserves, operations of the band government represent a disproportionately large amount of economic activity, and “government service” may have a different meaning than elsewhere. Compared to the female labour force of BC, the Aboriginal female labour force residing on reserves are more concentrated in the high-risk occupational categories of “trades, transport and equipment operators and related occupations”, and “occupations unique to primary industry. The Aboriginal female labour force is also more concentrated in the medium-risk category of “sales and service occupations”. Like Aboriginal males, the Aboriginal female labour force is more concentrated in the generally low-risk category of “occupations in social science, education, government service and religion.” By industry category, the Aboriginal male labour force is more concentrated in “agriculture, forestry, fishing and hunting”, “construction” and “manufacturing”. These are industries with relatively higher rates of worker compensation claims. The Aboriginal male labour force is also more concentrated in “mining and oil and gas extraction” (medium-risk), and “public administration”, and industry with a relatively low rate of worker compensation claims [22]. Again, on Aboriginal reserves, operations of the band government represent a disproportionately large amount of economic activity, and “public administration” may have a different meaning than elsewhere. The Aboriginal female labour force is more concentrated in the high-risk industrial categories of “agriculture, forestry, fishing and hunting”, and “construction”. The Aboriginal female labour force is also more concentrated in “mining and oil and gas extraction” (medium-risk), and “public administration”, and industry with a relatively low rate of worker compensation claims [22].

Tables 8 and 9 show regression statistics from the preliminary regression models with a single independent (X) variable. “P” is the probability of the null hypothesis that R^2^ is equal to zero. If “P” was less than 0.05, then the independent variable was retained for subsequent multivariable regression analysis.

**Table 8:** Ecological analysis of worker compensation injury risk among BC Aboriginal communities, 1999–2008, Regression [1] statistics from models with one independent (X) variable

**X Variable**	**units**	**min**	**max**	**mean [2]**	**SD [2]**	**N**	**R**^**2**^	**B**	**SE**	**P**	**RR Ratio per SD [2]**	**L95CL**	**U95CL**
Census	1 year	2001	2006	2003.5	2.5	319	0.012	0.020	0.010	0.049	1.053	1.000	1.108
Income Per Capita 1000	$1,000	5.3	50.9	13.1	5.9	147	0.067	0.022	0.007	0.002	1.135	1.051	1.226
Income Score	1	32.6	108.1	60.2	12.7	147	0.087	0.010	0.003	0.000	1.142	1.064	1.226
High School	1%	0.0	116.7	55.7	17.4	261	0.021	0.004	0.002	0.018	1.080	1.013	1.150
University Degree	1%	0.0	34.3	3.9	5.8	261	0.040	0.016	0.005	0.001	1.098	1.038	1.161
Pop Per Room	1	0.30	1.11	0.53	0.11	260	0.069	−1.294	0.296	0.000	0.866	0.811	0.924
Need Major Repairs	1%	0.0	120.0	32.7	19.2	261	0.005	−0.002	0.002	0.267	0.963	0.900	1.030
Labour Force	1%	9.9	100.0	61.7	12.3	261	0.005	−0.003	0.003	0.235	0.959	0.894	1.028
Employed	1%	7.7	77.3	47.3	11.0	261	0.027	0.008	0.003	0.008	1.094	1.024	1.169
Occupation Risk	RR	0.00	2.71	1.12	0.36	261	0.008	0.148	0.105	0.159	1.054	0.979	1.135
Industry Risk	RR	0.00	3.92	1.11	0.34	261	0.005	0.157	0.141	0.265	1.054	0.961	1.157
Remoteness	1	0.08	1.35	0.23	0.22	317	0.008	−0.183	0.113	0.108	0.961	0.916	1.009
Environ Index	1	0.40	3.00	0.65	0.38	317	0.012	−0.133	0.068	0.051	0.950	0.902	1.000
Aboriginal	1%	5.7	100.0	84.7	23.2	261	0.060	−0.005	0.001	0.000	0.892	0.844	0.943
NAIndian	1%	5.6	103.1	81.5	23.8	261	0.054	−0.005	0.001	0.000	0.896	0.847	0.948

Notes:

1. The dependent (Y) variable is Ln (SRR of worker compensation injury, total of all injuries), and regression is weighted by person-years.

2. Unweighted mean and standard deviation.

**Table 9:** Ecological analysis of worker compensation injury risk among BC Aboriginal communities, 1999–2008, Regression [1] statistics from models with one independent (X) variable

**X Variable, Interaction term**	**units**	**min**	**max**	**mean [2]**	**SD [2]**	**N**	**R**^**2**^	**B**	**SE**	**P**	**RR Ratio per SD [2]**	**L95CL**	**U95CL**
Census_Employed	1 year	153.9	1550	947.2	220.4	261	0.027	0.000	0.000	0.007	1.095	1.025	1.169
IncomePerCapita1000_Employed	$1,000	1.2	39.0	6.1	4.1	147	0.061	0.032	0.010	0.003	1.139	1.047	1.238
IncomeScore_Employed	1	5.5	82.7	27.7	10.4	147	0.089	0.014	0.004	0.000	1.153	1.070	1.243
HighSchool_Employed	1%	0.0	72.9	27.1	12.2	261	0.026	0.007	0.003	0.009	1.091	1.022	1.165
UniversityDegree_Employed	1%	0.0	26.3	2.0	3.5	261	0.038	0.028	0.009	0.002	1.102	1.038	1.171
PopPerRoom_Employed	1	0.03	0.47	0.25	0.07	260	0.000	−0.146	0.470	0.756	0.990	0.927	1.056
NeedMajorRepairs_Employed	1%	0.0	60.0	15.4	9.3	261	0.001	0.002	0.004	0.574	1.020	0.951	1.094
LabourForce_Employed	1%	0.8	66.7	30.2	11.4	261	0.004	0.003	0.003	0.311	1.036	0.967	1.109
OccupationRisk_Employed	RR	0.00	1.34	0.52	0.19	261	0.041	0.663	0.200	0.001	1.134	1.052	1.222
IndustryRisk_Employed	RR	0.00	1.12	0.52	0.17	261	0.030	0.631	0.224	0.005	1.115	1.033	1.204
Remoteness_Employed	1	0.01	0.75	0.11	0.10	261	0.000	−0.044	0.246	0.859	0.995	0.947	1.047
EnvironIndex_Employed	1	0.03	1.41	0.30	0.18	261	0.001	−0.076	0.145	0.601	0.986	0.936	1.039
Aboriginal_Employed	1%	1.1	75.0	40.2	13.9	261	0.008	−0.003	0.002	0.150	0.956	0.899	1.016
NAIndian_Employed	1%	1.0	68.8	38.8	14.0	261	0.007	−0.003	0.002	0.169	0.958	0.900	1.019

Notes:

1. The dependent (Y) variable is Ln (SRR of worker compensation injury, total of all injuries), and regression is weighted by person-years.

2. Unweighted mean and standard deviation.

Table 10 shows regression statistics from the best-fitting regression model with multiple independent (X) variables. The best-fitting model identified the following as statistically significant predictors of worker compensation injury risk: population per room, proportion of the population who identified themselves as Aboriginal, income score multiplied by employment, occupational risk multiplied by employment, proportion of university educated persons multiplied by employment, and Census year multiplied by employment. The entire model explained 32.5% of the variance among communities in SRR of worker compensation injury (R^2^ = 0.325, p < 0.0005).

**Table 10:** Ecological analysis of worker compensation injury risk among BC Aboriginal communities, 1999–2008, Regression [1] statistics from the best-fitting model with multiple independent (X) variables

**X Variable**	**units**	**min**	**max**	**mean [2]**	**SD [2]**	**N**	**B**	**SE**	**P**	**RR Ratio per SD [2]**	**L95CL**	**U95CL**
(Constant)						147	0.285	0.282	0.313			
PopPerRoom	1	0.30	1.11	0.53	0.11	147	−1.878	0.528	0.001	0.811	0.722	0.911
Aboriginal	1%	5.7	100.0	84.7	23.2	147	−0.007	0.002	0.000	0.847	0.777	0.923
IncomeScore_Employed	1	5.5	82.7	27.7	10.4	147	−0.048	0.012	0.000	0.606	0.472	0.777
OccupationRisk_Employed	RR	0.00	1.34	0.52	0.19	147	1.801	0.369	0.000	1.407	1.225	1.615
UniversityDegree_Employed	1%	0.0	26.3	2.0	3.5	147	0.050	0.014	0.001	1.189	1.077	1.313
Census_Employed	1 year	154	1550	947.2	220.4	147	0.002	0.000	0.002	1.395	1.133	1.717

Notes:

Multivariable model statistics: R^2^ = 0.325, F = 11.232, p = 0.000

1. The dependent (Y) variable is Ln (SRR of worker compensation injury, total of all injuries), and regression is weighted by person-years.

2. Unweighted mean and standard deviation.

## Discussion

It has been asserted that Aboriginal people in BC are at higher risk of injury than the total population, but our descriptive statistics offer a more varied perspective. In the category of worker compensation injury, Aboriginal people generally have *lower* risk. There are exceptions: in some HSDAs, and among women aged 50 years and older, Aboriginal people are at higher risk. Disparities in worker compensation injury risk might result from the competing effects of employment rates, occupations and industries. Aboriginal people have lower employment rates than the general population, but are more likely to work in hazardous occupations and industries. During the period 1987–2010, worker compensation injury rates declined for both the Aboriginal and the total populations, probably reflecting a secular trend towards safer work environments, but the decline was less among Aboriginal people. During the economic “boom” (measured in 2002 dollars, during the 5 years from 2002 to 2007, the Gross Domestic Product (GDP) of BC grew 19.0%, a year-over-year average of 3.5%), risk of worker compensation injury increased among Aboriginal people and went higher than the risk among the total population. In contrast, during the subsequent economic “bust” (during the two years from 2007 to 2009, GDP shrank 1.7%) [24], risk among Aboriginal people declined sharply to below the level of risk among the total population. As shown by our own analysis of Census data (see above), between the census years 2001 and 2006 the employment rate among Aboriginal reserve residents increased, and so did the hazardousness of their occupations. The jobs were insecure, because economic fluctuations were more severe in the industrial sectors where Aboriginal workers are concentrated. In “agriculture, forestry, fishing and hunting”, measured in 2002 dollars, during the 5 years from 2002 to 2007, the GDP grew by 4.3%, then in the subsequent two years from 2007 to 2009, GDP shrank disastrously by 16.2%. During the same periods respectively, in “construction” the GDP grew by an astonishing 43.8% then shrank markedly by 5.3% [24].

Our ecological analysis of hypothesized socio-economic, work-related, and geographic risk markers demonstrates some interesting associations, and may provide clues regarding the web of causation surrounding risk of worker compensation injury among the Aboriginal population.

The best-fitting model indicated that increased household population per room, and increased proportion of the population who identified as Aboriginal were associated with *decreased* risk of injury. We are accustomed to associating crowded housing and Aboriginal ethnicity with socio-economic disadvantage. However, in a multivariable model (controlling for employment, occupation, income and education), population per room and Aboriginal identity may reflect family structure and cultural adherence, rather than disadvantaged economic conditions. It is plausible that living in communities where people value extended family relationships and have strong identification with Aboriginal heritage could have psychological benefits that lower the risk of injury among community members [5].

In the descriptive, individual-level analysis, we observed that among the Aboriginal population, urban residents were at higher risk of worker compensation injury than Aboriginal people who were not urban. But the ecologic analysis tested two geographic variables, “remoteness” and “environmental index” that were derived from distance to the nearest urban centre, and neither was independently associated with risk of worker compensation injury. This suggests that the higher risk among urban residents is due to intervention by one or some combination of the variables retained in the final model. Likely the variable was “employment”, and it is plausible that urban dwelling Aboriginal people are more at risk for worker compensation injury because they are more likely to be employed.

The best-fitting model included the proportion of the population who were employed, but not as an independent variable with a directly proportional association with injury risk. Employment interacts multiplicatively with income, occupational risk, and university education. Increased occupational risk and increased employment, interacting together multiplicatively, are strongly associated with increased risk of worker compensation injury. Increased income and increased employment, interacting together multiplicatively, are strongly associated with *decreased* risk of worker compensation injury. These findings are plausible, as well as empirical. But it seems paradoxical that increased proportion of the population who are university-educated, and increased employment, interacting together multiplicatively, are associated with *increased* risk of worker compensation injury. Perhaps this indicates that university-educated people can better access the worker compensation system. Alternatively, this may indicate that among Aboriginal people, having university education may lead to mismatching of educational level with job category, increasing the risk of worker compensation injury. Or, the paradox may be ecological: increased proportion with university education among the population may indicate a more unequal social order, with increased injury risk to those in the lower strata.

Time (as measured by Census year) and increased employment, interacting together multiplicatively, are associated with increasing risk of worker compensation injury. This is disturbing, yet intriguing, as it suggests that among Aboriginal communities, there are other time-related factors that we have not measured, that are pushing worker compensation injury rates upwards, or preventing them from declining as much as injury rates in the total population.

Our ecological multivariable analysis studied only Aboriginal communities. We did not include any non-Aboriginal communities. Therefore, the findings only apply to Aboriginal communities, and cannot be used to explain the observed differences in worker compensation injury rates between the Aboriginal and total populations of BC. This matter invites future research, that includes both Aboriginal and non-Aboriginal communities in an ecological analysis.

### Data quality

BC’s universal health care insurance program is the best available registry of the province’s population. Using this registry, in fiscal year 2006–2007 we counted 4,266,070 people in BC, which is 103.7% of the number (4,113,487) enumerated in BC by the 2006 Census of Canada. The slight excess may represent persons who were deceased or no longer resident in the province, but who had not yet been removed from the insurance registry.

Using the insurance registry and our definition of “Aboriginal” (derived from insurance premium group and notations on birth and death records), in fiscal year 2006–2007 we counted 148,458 people in BC whom we considered “Aboriginal”, which is 75.8% of the number (196,070) enumerated in BC who identified themselves as “an Aboriginal person, that is, North American Indian, Métis or Inuit (Eskimo)” in the 2006 Census of Canada. Our definition of “Aboriginal” is admittedly restrictive, and largely, if indirectly, based on legally recognized Indian status, as defined by the Indian Act of Canada. Some might say that we should have determined Aboriginality using the federal government’s Indian Status Registry, but due to privacy issues and political considerations, it was not possible for us to get access to the Indian Status Registry. However, we consider our definition of “Aboriginal” to be superior to presence in the Indian Status Registry, because our definition includes residence in BC, whereas the Indian Status Registry reflects membership in a recognized First Nation or Indian band located in BC, regardless of where the individual in fact resides. Also, our definition is more likely to include children who are eligible for Indian status because of their parents’ Indian status, but who have not yet applied to be included in the Indian Status Registry.

We counted injuries registered for claims with the provincial worker compensation system. Work Safe BC’s database is the reference standard. There is no better. We have confidence in its accuracy because compensation payments depend on this database, and people who do not get the payments to which they are entitled will take action to claim their due. Some may argue that limiting our analysis to injuries registered for worker compensation claims imposes an overly restrictive definition of occupational injury. However, limiting our definition helps to protect the internal validity of our analysis.

## Conclusions

As an increasing proportion of Aboriginal people became employed with pay, over the past decade incidence of worker compensation injury among the Aboriginal population has reached parity with, or even exceeded that among the general population. We need culturally sensitive workplace injury prevention programming, particularly in geographic regions and industries where Aboriginal workers are concentrated. Targets for prevention programs should include older Aboriginal people, especially women. It is conventional wisdom that employment is good for health, but our analysis suggests the effects may be mixed. This challenge can be met with further knowledge and better-informed planning.

## Abbreviations

BC: British Columbia; GDP: Gross Domestic Product; HSDA: Health Service Delivery Area; MSP: Medical Services Plan of British Columbia; SRR: Standardized Relative Risk.

## Competing interests

Andrew Jin, M. Anne George, Mariana Brussoni, and Christopher E. Lalonde declare that they have no competing interests.

## Authors’ contributions

AJ participated in the conception and design of the study, performed the statistical analysis and drafted the manuscript. MAG participated in the conception and design of the study and edited the manuscript. MB participated in the conception and design of the study and edited the manuscript. CEL participated in the conception and design of the study and edited the manuscript. All authors read and approved the final manuscript.

## Authors’ information

AJ is self-employed as an epidemiology consultant. MAG is an Associate Professor in the Department of Pediatrics, Faculty of Medicine, University of British Columbia, and Scientist Level 1 at the Child and Family Research Institute. MB is an Assistant Professor in the Department of Pediatrics, Faculty of Medicine, University of British Columbia, and Scientist Level 1 at the Child and Family Research Institute. CEL is a Professor in the Department of Psychology, Faculty of Social Sciences, University of Victoria.

## Pre-publication history

The pre-publication history for this paper can be accessed here:

http://www.biomedcentral.com/1471-2458/14/710/prepub
